# Axillary response according to neoadjuvant single or dual human epidermal growth factor receptor 2 (HER2) blockade in clinically node‐positive, HER2‐positive breast cancer

**DOI:** 10.1002/ijc.33726

**Published:** 2021-07-08

**Authors:** Chihwan Cha, Sung Gwe Ahn, Dooreh Kim, Janghee Lee, Soeun Park, Soong June Bae, Jee Ye Kim, Hyung Seok Park, Seho Park, Seung Il Kim, Byeong‐Woo Park, Joon Jeong

**Affiliations:** ^1^ Department of Surgery Hanyang University College of Medicine Seoul South Korea; ^2^ Department of Surgery Gangnam Severance Hospital, Yonsei University College of Medicine Seoul South Korea; ^3^ Institute for Breast Cancer Precision Medicine Yonsei University College of Medicine Seoul South Korea; ^4^ Department of Surgery Dongtan Sacred Heart Hospital, Hallym University Hwaseong South Korea; ^5^ Department of Surgery CHA Ilsan Medical Center, CHA University School of Medicine Goyang‐si South Korea; ^6^ Department of Surgery Severance Hospital, Yonsei University College of Medicine Seoul South Korea

**Keywords:** axillary response, HER2‐positive breast cancer, neoadjuvant therapy, pertuzumab, trastuzumab

## Abstract

Incorporating dual human epidermal growth factor receptor 2 (HER2) blockade into neoadjuvant systemic therapy (NST) led to higher response in patients with HER2‐positive breast cancer. However, axillary response to treatment regimens, including single or dual HER2 blockade, in patients with clinically node‐positive breast cancer remains uncertain. Our study aimed to examine the pathologic axillary response according to the type of NST, that is, single or dual HER2 blockade. In our study, 546 patients with clinically node‐positive, HER2‐positive breast cancer who received NST followed by axillary surgery were retrospectively selected and divided into three groups: chemotherapy alone, chemotherapy + trastuzumab and chemotherapy + trastuzumab with pertuzumab. The primary outcome was the axillary pathologic complete response (pCR). Among 471 patients undergoing axillary lymph node dissection, the axillary pCR rates were 43.5%, 74.5% and 68.8% in patients who received chemotherapy alone, chemotherapy + trastuzumab and chemotherapy + trastuzumab with pertuzumab, respectively. There was no difference in axillary pCR rates between patients who received single or dual HER2 blockade (*P* = .379). Among patients receiving chemotherapy + trastuzumab, patients without breast pCR had the greatest risk for residual axillary metastases (relative risk, 9.8; 95% confidence interval, 3.2‐14.9; *P* < .0001). In conclusion, adding trastuzumab to chemotherapy increased the axillary pCR rate in patients with clinically node‐positive, HER2‐positive breast cancer; furthermore, dual HER2‐blockade with trastuzumab and pertuzumab did not elevate the axillary response compared with trastuzumab alone. Breast pCR could be a strong predictor for axillary pCR in clinically node‐positive patients treated with HER2‐targeting therapy.

## Abstract 2

What's new?

Dual blockade of the human epidermal growth factor receptor 2 (HER2) with trastuzumab and pertuzumab is the preferred option for neoadjuvant systemic therapy (NST) against node‐positive, HER2‐positive breast cancer. Whether this approach effectively improves pathologic complete response (pCR), however, remains unclear. Here, comparison of pCR for single or dual HER2 blockade shows that trastuzumab effectively increases axillary pCR rate in clinically node‐positive, HER2‐positive breast cancer. The addition of pertuzumab to trastuzumab did not further elevate pCR rate. The findings are relevant to decisions regarding axillary surgery in breast cancer patients with pCR after NST with HER2 targeted agents.

**Abbreviations**
ADCC: antibody‐dependent cellular cytotoxicity
ALND: axillary lymph node dissection
BCS: breast‐conserving surgery
ER: estrogen receptor
H: trastuzumab
HER2: human epidermal growth factor receptor 2
HP: trastuzumab and pertuzumab
NPV: negative predictive value
NST: neoadjuvant systemic therapy
OR: odds ratio
pCR: pathologic complete response
RR: relative risk
SLNB: sentinel lymph node biopsy
SNs: sentinel nodes
TNBC: triple‐negative breast cancer

## INTRODUCTION

For downstaging primary tumors, neoadjuvant systemic therapy (NST) has been commonly used for managing patients with locally advanced breast cancer. Moreover, robust clinical evidence suggests that patients with pathologic complete response (pCR) after NST had a superior survival outcome compared to those with non‐pCR at an individual level.^1^ Although a higher pCR rate did not automatically suggest a better survival at the trial level, NST has been the preferred option for managing human epidermal growth factor receptor 2 (HER2)‐positive subtype or triple‐negative breast cancer (TNBC).^2^, ^3^, ^4^

Responses to NST highly depend on tumor biology and thus differ according to the tumor subtype. The pCR rates are higher in HER2‐positive cancer and TNBC than in other subtypes.^5^, ^6^ Although no specific target therapy is currently available for early TNBC outside clinical trials, the development and application of HER2‐targeted drugs have improved the efficacy of NST for HER2‐positive breast cancer.^7^, ^8^ Specifically, incorporating dual HER2‐targeted drugs into NST has led to a higher pathologic response in patients with HER2‐positive breast cancer.^9^ Since dual HER2 blockade with trastuzumab and pertuzumab has been implemented in NST, the pCR rate has increased up to approximately 60%.^10^, ^11^, ^12^ Additionally, a previous prospective trial demonstrated a clinical benefit of adding pertuzumab to chemotherapy and trastuzumab as adjuvant therapy, specifically for node‐positive HER2 breast cancer.^13^ Currently, dual HER2 blockade with trastuzumab and pertuzumab is the preferred anti‐HER2 treatment option for node‐positive, HER2‐positive breast cancer.^14^

Considering the surgeons' viewpoint, a higher response to NST may considerably reduce the extent of axillary surgery in patients with clinically node‐positive breast cancer. Several clinical trials evaluated the feasibility of sentinel lymph node biopsy (SLNB) after NST in patients with initial axillary metastases.^15^, ^16^ These trials commonly suggest that SLNB could not replace axillary lymph node dissection (ALND) in all comers of node‐positive patients at initial presentation but could be an alternative option when three or more negative sentinel nodes (SNs) were detected and all were proven to be negative after a pathologic evaluation.^17^, ^18^, ^19^ Based on these findings, recent guidelines recommend SLNB instead of upfront ALND in selected patients with conversion to node‐negative disease after NST. However, clinical reports of nodal response after neoadjuvant dual anti‐HER2 blockade remain insufficient for node‐positive HER2‐breast cancer despite the addition of dual HER2‐targeted therapy to the armamentarium against HER2 breast cancer.

In our study, we examined the pathologic axillary response according to the types of NST, that is, single or dual HER2 blockade, in 546 consecutive patients. Additionally, the association between axillary pCR and breast pCR for different treatment regimens was analyzed.

## 1 MATERIALS AND METHODS

### 1.1 Patients

We retrospectively reviewed patients' medical records from two institutions (Gangnam Severance Hospital and Severance Hospital) from 2007 to 2018; 546 patients with clinically node‐positive, HER2‐positive breast cancer treated with NST were included. All patients had clinically axillary nodal metastasis before neoadjuvant treatments and underwent proper axillary surgery with or without SLNB after completing NST. Patients who discontinued chemotherapy because of disease progression or toxicity were excluded.

HER2 status (positive or negative) was defined based on immunohistochemical analysis and was confirmed using fluorescence in situ hybridization according to the American Society of Clinical Oncology/College of American Pathologists guidelines.^20^ Clinical and pathologic data of 546 patients were identified from the medical database.

Axillary nodal metastasis before chemotherapy was diagnosed by physical examination and radiologic modalities, including ultrasonography and magnetic resonance imaging. Among 546 patients, 376 patients (68.9%) had pathologically proven metastatic lymph node confirmed by fine‐needle aspiration or core‐needle biopsy. Details regarding neoadjuvant treatment regimens were collected. Patients were divided into three groups based on whether they received single or dual HER2‐targeted therapy: chemotherapy alone, chemotherapy with trastuzumab (H) and chemotherapy with trastuzumab and pertuzumab (HP). Further information regarding the regimens is provided in Table S1. Gangnam Severance Hospital Institutional Review Board approved the study, and a waiver for obtaining informed consent was granted based on the retrospective nature of the study.

### 1.2 Axillary surgery

After completing NST, all patients underwent breast surgery (mastectomy or breast‐conserving surgery [BCS]) with proper axillary surgery (SLNB alone, ALND after SLNB or ALND alone) (Figure S1). Before 2012, most patients underwent upfront ALND or backup ALND after SLNB. Since the results of the prospective studies ACOSOG‐Z1071 and SENTINA were published, ALND was omitted if at least three or more SNs were detected and all were proven to be negative after pathologic evaluation.

SLNB was successfully performed in 361 patients. For SLN mapping, we preferred a radiolabeled colloid (technetium‐99 m) as a single tracer injected in peri‐areolar sites.^21^ However, a dual tracer with blue dye and a radiolabeled colloid were used in 36 patients (10.0%). Lymph nodes with more than 10% of the hottest node's radioactivity or stained with blue dye were considered SNs and resected. The type of surgery performed was based on the surgeon's discretion.

### 1.3 Pathologic evaluation

The pathologic response to NST was defined as a breast pCR if there was no evidence of residual invasive tumor in the breast and as an axillary pCR if there was no evidence of residual tumor cells in resected axillary lymph nodes. Total pCR was defined as the achievement of both breast and axillary pCRs. The pathologic status of axillary lymph nodes was determined based on the examination of hematoxylin and eosin‐stained sections from each block of serially sectioned lymph nodes.

### 1.4 Statistical analyses

The primary outcome was the axillary pCR rate of each NST regimen. The axillary pCR rate was compared between different treatment regimens and patients who underwent ALND. The secondary outcome was the association between axillary pCR and breast pCR according to the treatment regimen. The negative predictive value (NPV) of breast pCR for concomitant axillary pCR was calculated for each treatment regimen to assess the association between axillary pCR and breast pCR. The incidence of pathologic nodal metastases was calculated for patients with and without breast pCR and compared using relative risk (RR) ratios with 95% confidence intervals (CIs). As the statistical differences of axillary pCR rates according to treatment regimens were similar across the cohort and in patients who underwent ALND, NPV and RR were analyzed only for the cohort.

Data were analyzed using the Statistical Package for the Social Sciences version 23.0. Clinicopathologic characteristics of the groups were compared using chi‐squared analysis and Mann‐Whitney *U* test. We used a logistic regression model to identify predictive factors for axillary pCR. The statistical significance level (*P*) was taken as a measure of the strength of evidence, and *P* < .05 was considered statistically significant.

## 2 RESULTS

### 2.1 Patient characteristics

Among 546 patients in our study, 285 (52.2%) were treated with chemotherapy alone; 134 (24.5%) with chemotherapy and trastuzumab (H) and 127 (23.3%) with chemotherapy, trastuzumab and pertuzumab (HP). The majority of patients (68.4%) in the chemotherapy alone group underwent surgery in the early period (from 2007 to 2011).

Among all patients, 333 patients (61.0%) achieved axillary pCR after NST and 261 patients (47.8%) achieved breast pCR. Moreover, 223 patients (40.8%) achieved total pCR. Patient and tumor characteristics according to neoadjuvant treatment regimens are summarized in Table 1. There were no differences between study groups with respect to age, estrogen receptor (ER) status and clinical nodal stage at diagnosis. Initial tumor size was larger in the HP subgroup than in the other groups. The rates of BCS were not different among the study groups, whereas the rate of ALND regardless of SLNB was the highest in the group preoperatively treated with chemotherapy alone (*P* < .001).

**TABLE 1:** Clinical and pathologic characteristics

Characteristics	Study groups			
	Chemotherapy only (N = 285)	Chemotherapy + H (N = 134)	Chemotherapy + HP (N = 127)	*P* value
Age (y), median (range)	50 (26‐70)	51 (27‐76)	48 (22‐75)	.069
ER status				.071
Positive	150 (52.6)	61 (45.5)	52 (40.9)	
Negative	135 (47.4)	73 (54.5)	75 (59.1)	
Initial T size (cm), median (range)	3.2 (0.7‐10.0)	3.5 (0.8‐10.0)	3.9 (1.5‐10.7)	.004
Initial nodal stage				.816
cN1	207 (72.6)	101 (75.4)	92 (72.4)	
cN2/cN3	78 (27.4)	33 (24.6)	35 (27.6)	
Clinical stage at diagnosis				.580
II	179 (62.8)	80 (60.2)	73 (57.5)	
III	106 (37.2)	53 (39.8)	54 (42.5)	
Breast surgery				.085
BCS	93 (32.6)	58 (43.6)	43 (34.1)	
Total mastectomy	192 (67.4)	75 (56.4)	83 (65.9)	
Axillary surgery				<.001
SLNB only	7 (2.5)	21 (15.7)	47 (37.0)	
ALND after SLNB	174 (61.1)	54 (40.3)	58 (45.7)	
ALND only	104 (36.5)	59 (44.0)	22 (17.3)	

Abbreviations: ALND, axillary lymph node dissection; BCS, breast‐conserving surgery; ER, estrogen receptor; H, trastuzumab; HP, trastuzumab and pertuzumab; SLNB, sentinel lymph node biopsy.

### 2.2 Breast and axillary pCR rates according to preoperative treatment regimen

**FIGURE 1:**
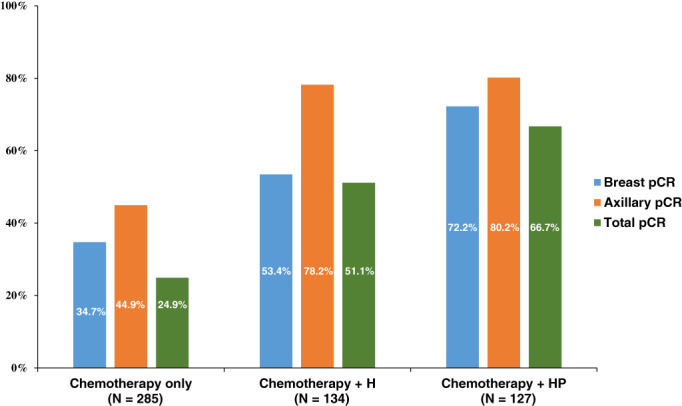
Breast and axillary pathologic complete response rates according to neoadjuvant treatment regimens. Blue, orange and green bars indicate breast, axillary and total pCR rates, respectively. H, trastuzumab; HP, trastuzumab and pertuzumab; pCR, pathologic complete response [Color figure can be viewed at wileyonlinelibrary.com]

Breast and axillary pCR rates according to treatment regimen are shown in Figure 1. The axillary and breast pCR rates were 44.9%, 78.2% and 80.2%, and 34.7%, 53.4% and 72.2% in patients in the chemotherapy alone group, H group and HP group, respectively (*P* < .001, *P* < .001). The breast pCR rate was higher in the group treated with a dual HER2 blockade than in the group treated with a single HER2 blockade (*P* = .002). However, there was no difference in the axillary pCR rates between the two groups (trastuzumab with or without pertuzumab) (*P* = .778). The axillary pCR rates were higher than the breast pCR rates for all treatment regimens, and the difference between the axillary and breast pCR rate was the greatest in the group receiving trastuzumab (24.8%, *P* < .001). The addition of trastuzumab to chemotherapy led to a higher axillary pCR rate by 1.7‐fold and a higher breast pCR rate by 1.5‐fold compared with chemotherapy alone. However, the addition of pertuzumab to a trastuzumab‐containing regimen increased the breast pCR rate but did not increase the axillary pCR rate.

A sensitivity analysis was performed in patients with biopsy‐proven axillary metastasis. As shown in Figure S2, axillary pCR rates were 43.2%, 76.6% and 73.3% in the chemotherapy alone group, H group and HP group, respectively (*P* < .001). There was no difference in the axillary pCR rates between the H and HP groups (*P* = .610); after stratifying by clinical nodal stage, there was still no difference in the axillary pCR rates between both the groups.

### 2.3 Axillary pCR in patients undergoing ALND

Regarding axillary surgery, 361 patients (66.1%) underwent SLNB and 471 patients (86.3%) underwent ALND. Among 471 patients undergoing ALND, the axillary pCR rates were 43.5%, 74.5% and 68.8% in the chemotherapy alone, H and HP groups, respectively (*P* < .001). There was no difference in the axillary pCR rates according to single or dual HER2 blockade (*P* = .379). Among 286 patients undergoing ALND after SLNB, 197 patients underwent ALND despite negative SLNB results. In this subgroup, the axillary pCR rates were 72.6%, 89.1% and 87.6% in the chemotherapy alone, H and HP groups, respectively (*P* = .028). There was no difference in the axillary pCR rates according to single or dual HER2 blockade (*P* = .718). Among 104 patients with more than three sentinel lymph nodes retrieved, the axillary pCR rates indicating the candidates for omission of ALND were 39.4%, 75.0% and 72.7% in the chemotherapy alone, H group and HP group, respectively (*P* = .003). There was no difference in the axillary pCR rates according to single or dual HER2 blockade (*P* = .875).

### 2.4 Predictors of axillary pCR

According to a univariate analysis, clinical N1 stage at diagnosis, chemotherapy with H or HP, breast pCR and ER negativity were found to be significant predictors of axillary pCR. Moreover, according to a multivariate logistic regression analysis, breast pCR was the most significant predictor for axillary pCR (odds ratio [OR], 7.187; 95% CI, 4.586‐11.264; *P* < .001). Additionally, chemotherapy with H (OR, 4.164; 95% CI, 2.457‐7.058; *P* < .001), chemotherapy with HP (OR, 3.172; 95% CI, 1.788‐5.628; *P* < .001), clinical N1 stage at diagnosis (OR, 1.755; 95% CI, 1.089‐2.826; *P* = .021) and ER negativity (OR, 1.652; 95% CI, 1.080‐2.526; *P* = .021) were significant predictors for axillary pCR after adjusting age and tumor size (Table 2).

**TABLE 2:** Univariate and multivariate analyses to determine predictors of axillary pathologic complete response

Variables	Univariate analysis		Multivariate analysis	
	OR (95% CI)	*P* value	OR (95% CI)	*P* value
Age (y)^a^	1.017 (0.998‐1.036)	.079	**—**	**—**
Initial tumor size (cm)^a^	0.970 (0.891‐1.055)	.474	**—**	**—**
**Initial nodal stage**		.008		.021
Clinical N2/N3 stage	Reference		Reference	
Clinical N1 stage	1.608 (1.131‐2.286)		1.755 (1.089–2.826)	
**Treatments**				
Chemotherapy	Reference		Reference	
Chemotherapy with H	2.498 (1.948‐3.204)	<.001	4.164 (2.457–7.058)	<.001
Chemotherapy with HP	3.131 (1.953‐5.020)	<.001	3.172 (1.788–5.628)	<.001
**Breast pCR**		<.001		<.001
Non‐pCR	Reference		Reference	
pCR	9.368 (6.161‐14.245)		7.187 (4.586–11.264)	
**Estrogen receptor**		<.001		.021
Positive	Reference		Reference	
Negative	2.139 (1.507‐3.036)		1.652 (1.080–2.526)	

Abbreviations: CI, confidence interval; H, trastuzumab; HP, trastuzumab and pertuzumab; OR, odds ratio; pCR, pathologic complete response.

^a^ Age and initial tumor size were considered continuous variables.

### 2.5 Association between breast and axillary pCR according to treatment regimens

**FIGURE 2:**
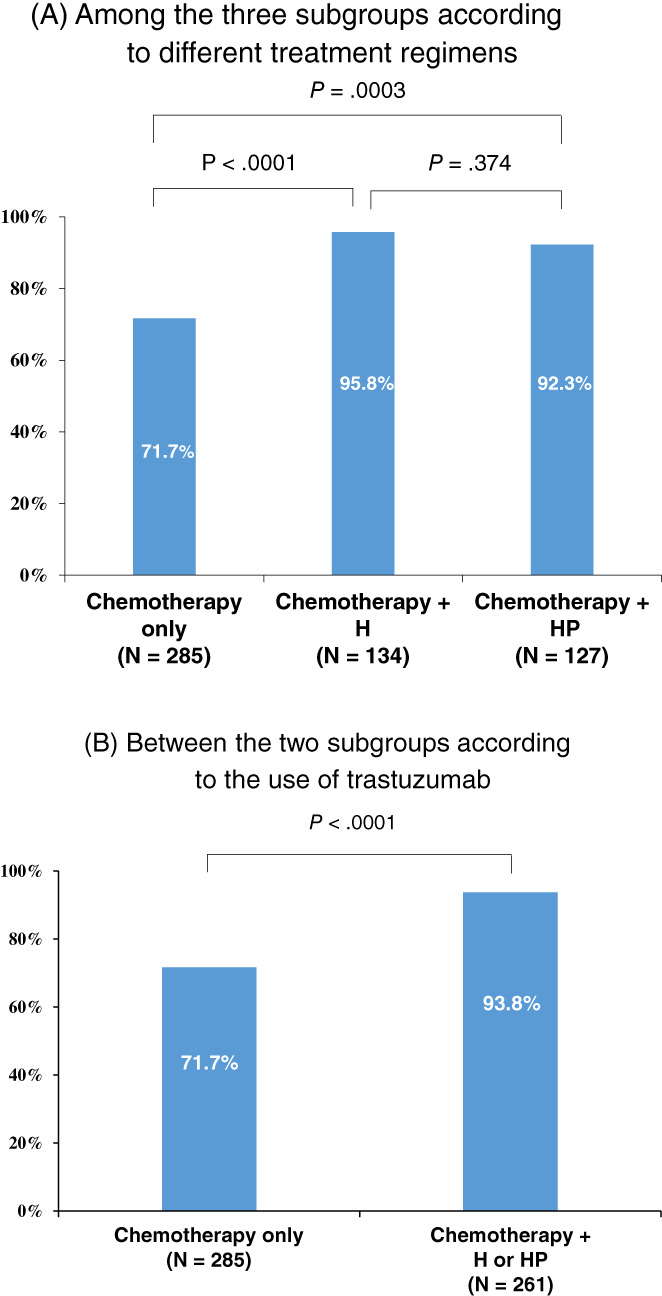
Positive predictive value of breast pathologic complete response (pCR) for concomitant axillary pCR according to treatment regimens. A, Among the three subgroups according to different treatment regimens. B, Between the two subgroups according to the use of trastuzumab. H, trastuzumab; HP, trastuzumab and pertuzumab [Color figure can be viewed at wileyonlinelibrary.com]

Among 285 patients receiving chemotherapy alone, 28 had breast pCR only and 71 had total pCR (both breast and axillary pCRs, Table S2). The NPV of breast pCR for concomitant axillary pCR was 71.7% (71/[71 + 28] × 100). Among 134 patients receiving chemotherapy with H, three had breast pCR only and 68 had total pCR. The NPV of breast pCR for axillary pCR was 95.8% (68/[68 + 3] × 100). Among 127 patients receiving chemotherapy with HP, seven had breast pCR only and 84 had total pCR. The NPV of breast pCR for axillary pCR was 92.3% (84/[84 + 7] × 100). The NPV was significantly higher in the H group than in the chemotherapy alone group (*P* < .0001); however, there was no difference between the single and dual HER2 blockade groups (*P* = .374) (Figure 2). False‐negative rates for predicting axillary pCR with breast pCR were 17.7%, 10.3% and 26.9% in the chemotherapy alone, H and HP groups, respectively.

Table 3 shows the RR ratios comparing the incidence of residual axillary nodal metastases after NST between patients with and without breast pCR. Patients receiving chemotherapy with H had the greatest increase in RR for residual axillary metastases among patients without breast pCR (RR, 9.8; 95% CI, 3.1‐30.7; *P* < .0001). RRs for residual axillary metastases were 2.5 (95% CI, 1.8‐3.4; *P* < .0001), and 6.9 (95% CI, 3.2‐14.9; *P* < .0001) in patients receiving chemotherapy alone and chemotherapy with HP, respectively.

**TABLE 3:** Relative risks for residual axillary metastases after NST in patients without and with breast pCR according to treatment regimens

Treatment regimen	RR (95% CI)	*P* value
Chemotherapy alone	2.5 (1.8‐3.4)	<.0001
Chemotherapy with H	9.8 (3.1‐30.7)	<.0001
Chemotherapy with HP	6.9 (3.2‐14.9)	<.0001

Abbreviations: CI, confidence interval; H, trastuzumab; HP, trastuzumab and pertuzumab; NST, neoadjuvant systemic therapy; pCR, pathologic complete response; RR, relative risk.

## 3 DISCUSSION

Dual HER2 blockade with trastuzumab and pertuzumab has become the most preferred therapeutic option for patients with node‐positive, HER2‐positive breast cancer, with clinical trials demonstrating its superiority to single HER2 blockade in terms of pCR rate^9^, ^10^ and disease‐free survival.^13^ If the addition of pertuzumab to NST with trastuzumab increases the axillary pCR rate and subsequently increases the total breast and axillary pCR rates, dual HER2 blockade may further reduce the frequency of axillary surgery for patients with clinically node‐positive, HER2‐positive breast cancer. Thus, we wanted to determine whether dual HER2 blockade has more advantages than single HER2 blockade with regard to pathologic axillary response in node‐positive breast cancer. According to our results, we found that the axillary pCR rate was higher in patients with node‐positive, HER2‐positive breast cancer who received NST with trastuzumab and chemotherapy than in those who received NST with chemotherapy alone. However, dual HER2 blockade with trastuzumab and pertuzumab did not increase the axillary pCR rate. Correspondingly, it is inferred that increased total pCR rate by dual HER2 blockade might be mainly attributed to the improvement of breast pCR and not to that of axillary pCR, although breast pCR was recognized as a strong predictor for axillary pCR (Table 2).

Currently, few studies have compared axillary pCR directly between patients who received NST with or without trastuzumab. Our findings suggest that treatment with trastuzumab and chemotherapy improves the axillary pCR rate compared with treatment with chemotherapy alone in patients with clinically node‐positive breast cancer. The higher axillary response in patients receiving trastuzumab might be explained by the immune system activation associated with antibody‐dependent cellular cytotoxicity (ADCC).^22^ The activation of ADCC has been described as an important mechanism of action of trastuzumab.^23^, ^24^, ^25^ As the immune system activation would be significantly effective in the lymphoid tissue of metastatic axillary nodes, NST with trastuzumab could enhance the axillary response in patients with metastatic lymph nodes. The axillary pCR rate of 78.2% in the chemotherapy with trastuzumab group in our study is consistent with the rate reported in a previous study. According to Dominici et al's study of 109 patients with HER2‐positive breast cancer and axillary metastasis,^26^ the axillary pCR rate was 74% in patients receiving chemotherapy with trastuzumab, suggesting that our axillary pCR rate corresponding to single HER2 blockade is reliable.

The axillary pCR rate of dual HER2 blockade has not been widely studied in patients with node‐positive and HER2‐positive breast cancer. In the current study, dual HER2 blockade comprising pertuzumab and trastuzumab did not lead to higher axillary pCR rates compared with single HER2 blockade with trastuzumab alone. The multivariate analysis revealed that the OR of dual HER2 blockade was 3.172 (1.788‐5.628), which was numerically lower than that of single HER2 blockade (4.164 [2.457‐7.058]) for axillary pCR, supporting the hypothesis that the addition of pertuzumab did not increase nodal response. Additionally, it is noteworthy to emphasize that the difference between the axillary and breast pCR rates was significantly observed in the single HER blockade group (Figure 2). Taken together, the synergistic effect of pertuzumab with trastuzumab might be confined to increased breast pCR. This finding might be explained by the nonsynergistic effect of dual HER2 blockade on ADCC activation. The combination of trastuzumab and pertuzumab at receptor‐saturating concentrations did not enhance ADCC activity based on an in vitro analysis.^27^ It could be assumed that HER2‐binding saturation with trastuzumab might be sufficient to activate the maximum response of immune effector cells in vivo; hence, there was no difference between the axillary pCR rates of single and dual HER2 blockade. Further studies are needed to corroborate our findings.

Regarding the associations between breast pCR and axillary pCR, several studies have reported that breast pCR is highly predictive of axillary pCR, as in our study. Tadros et al reported that breast pCR was highly associated with axillary pCR after NST.^28^ In this cohort study of 527 consecutive patients with HER2‐positive or triple‐negative (T1/T2 and N0/N1) breast cancer who achieved breast pCR after NST, 185 of the 193 (95.9%) patients achieved axillary pCR. They suggested that the feasibility for the omission of axillary surgery could be assessed in highly selective patients with breast pCR in a future prospective trial. In our results, patients with initial clinical node metastasis receiving chemotherapy with trastuzumab had a higher NPV of breast pCR (95.8%) for concomitant axillary pCR than patients receiving chemotherapy alone. However, the addition of pertuzumab did not increase the NPV of breast pCR compared with single HER2 blockade. Moreover, the RR for residual axillary metastases after NST in patients without breast pCR was greater among patients receiving single HER2 blockade than among those receiving dual HER2 blockade. To our knowledge, this is the first study investigating the association between breast and axillary pCR according to NST regimen containing single or dual HER2 blockade agents.

The potential limitation of our study is its retrospective study design. To compare the axillary response between the chemotherapy only treatment regimen and single or dual HER2 blockade, our study included patients who underwent NST from 2007 to 2018; the expanded study period could result in a potential selection bias. Most patients in the chemotherapy only group were treated at the beginning of the study period, while patients with single or dual HER2 blockade were treated mostly at the end. Since two prospective trials evaluating the role of SLNB in node‐positive patients were announced in 2012,^15^, ^16^ the rate of ALND might be affected by the study period. Furthermore, the implications of these findings on surgical decisions might vary among institutions and individual surgeons; therefore, our end point was axillary pCR rather than the type of axillary surgery. Despite this limitation, most patients (86.3%) in our study underwent ALND, and all surgically resected nodes were evaluated.

Another limitation is that backbone chemotherapy regimens and the frequency of delivered HER2 targeting therapy were different between single‐ and dual‐HER2 blockade groups (Table S1). Sequential anthracylines and taxanes were used only in the single‐HER2 blockade group. In addition, fourth cycles of trastuzumab were delivered in the single HER2 blockade group and sixth cycles of HP were done in the dual group. Different frequencies and regimens of chemotherapy and HER2 targeted therapy could influence nodal pCR outcome. However, we primarily aimed to know axillary response according to the types of HER2 blockades which are most preferred in daily practice, this limitation raised by different backbone chemotherapy regimen could be acceptable.

Further studies are required to confirm the reproducibility of our findings in patients with clinically node‐positive, HER2‐positive breast cancer treated with NST. Currently, there is an ongoing prospective, multicenter, single‐arm trial (NCT 04101851) investigating the feasibility of omitting axillary surgery in triple‐negative or HER2‐positive patients with radiologic CR and confirmed breast pCR on lumpectomy after NST.^29^ This study is based on the same rationale shown in our findings, namely that in‐breast pCR is the best predictor for axillary nodal pCR.

In conclusion, adding trastuzumab to chemotherapy increased the axillary pCR rate in patients with clinically node‐positive, HER2‐positive breast cancer, whereas dual HER2‐blockade with trastuzumab and pertuzumab did not elevate the axillary response compared with trastuzumab alone. The association between breast pCR and axillary pCR was stronger in patients receiving trastuzumab than in those receiving chemotherapy alone. Our findings could be implicated in the future clinical trials which evaluate the oncologic safety of de‐escalating axillary surgery in patients with breast pCR after NST with HER2 targeted agents.

## CONFLICT OF INTEREST

The authors declare no potential conflict of interest.

## ETHICS STATEMENT

All procedures performed in this study involving human participants were in accordance with the ethical standards of the institutional and national research committee, as well as the 1964 Helsinki Declaration and its later amendments. The protocol was approved by the institutional review board of the Gangnam Severance Hospital (IRB No. 3‐2019‐0298), and a waiver for obtaining informed consent was granted based on the retrospective nature of the study.

## Supporting information

**Appendix S1**: Supporting Information

## Data Availability

The data sets generated and analyzed in the current study are available from the corresponding author upon reasonable request.
