# Supplementary material for: Axillary response according to neoadjuvant single or dual human epidermal growth factor receptor 2 (HER2) blockade in clinically node‐positive, HER2‐positive breast cancer
Source: Int J Cancer. 2021 Jul 8;149(8):1585–92. doi: 10.1002/ijc.33726 (PMC8457167; doi:10.1002/ijc.33726)
Supplement: Supplementary file 1 — Appendix S1: Supporting Information [file IJC-149-1585-s001.pdf]

Supplementary material

Axillary response according to neoadjuvant single or dual human epidermal growth factor receptor 2 (HER2) blockade in clinically node-positive, HER2-positive breast cancer

Chihwan Cha, Sung Gwe Ahn, Dooreh Kim, Janghee Lee, Soeun Park, Soong June Bae, Jee Ye Kim, Hyung Seok Park, Seho Park, Seung Il Kim, Byeong-Woo Park, and Joon Jeong

Table of contents:

- Supplementary table 1
- Supplementary table 2
- Supplementary figure 1
- Supplementary figure 2

Table S1. Detailed information of treatment regimens

Neoadjuvant regimen	Chemotherapy only (N = 285)	Chemotherapy + H (N = 134)	Chemotherapy + HP (N = 127)
ACT	166 (58.2)		
AT	108 (37.9)		
CAF	11 (3.9)		
ACTH		121 (90.3)	
TCH		13 (9.7)	
TCHP			127 (100.0)

Abbreviations: H, trastuzumab; HP, trastuzumab and pertuzumab; ACT, 4 cycles of doxorubicin and cyclophosphamide and 4 cycles of taxane; AT, 6 cycles of doxorubicin and taxane; CAF, 6 cycles of cyclophosphamide and doxorubicin and 5-fluorouracil; ACTH, 4 cycles of doxorubicin and cyclophosphamide and 4 cycles of taxane and trastuzumab; TCH, 6 cycles of taxane and carboplatin and trastuzumab; TCHP, 6 cycles of taxane and carboplatin and trastuzumab and pertuzumab

Table S2. Breast and/or axillary pathologic complete response according to treatment regimens

(a) Patients receiving chemotherapy alone (N = 285)

	Breast pCR	Residual breast lesion
Axillary pCR	71	56
Residual axillary metastasis	28	130

(b) Patients receiving chemotherapy + H (N = 134)

	Breast pCR	Residual breast lesion
Axillary pCR	68	37
Residual axillary metastasis	3	26

(a) Patients receiving chemotherapy + HP (N = 127)

	Breast pCR	Residual breast lesion
Axillary pCR	84	17
Residual axillary metastasis	7	19

Abbreviations: H, trastuzumab; HP, trastuzumab and pertuzumab

Figure S1. Consort flow diagram of patients in the study

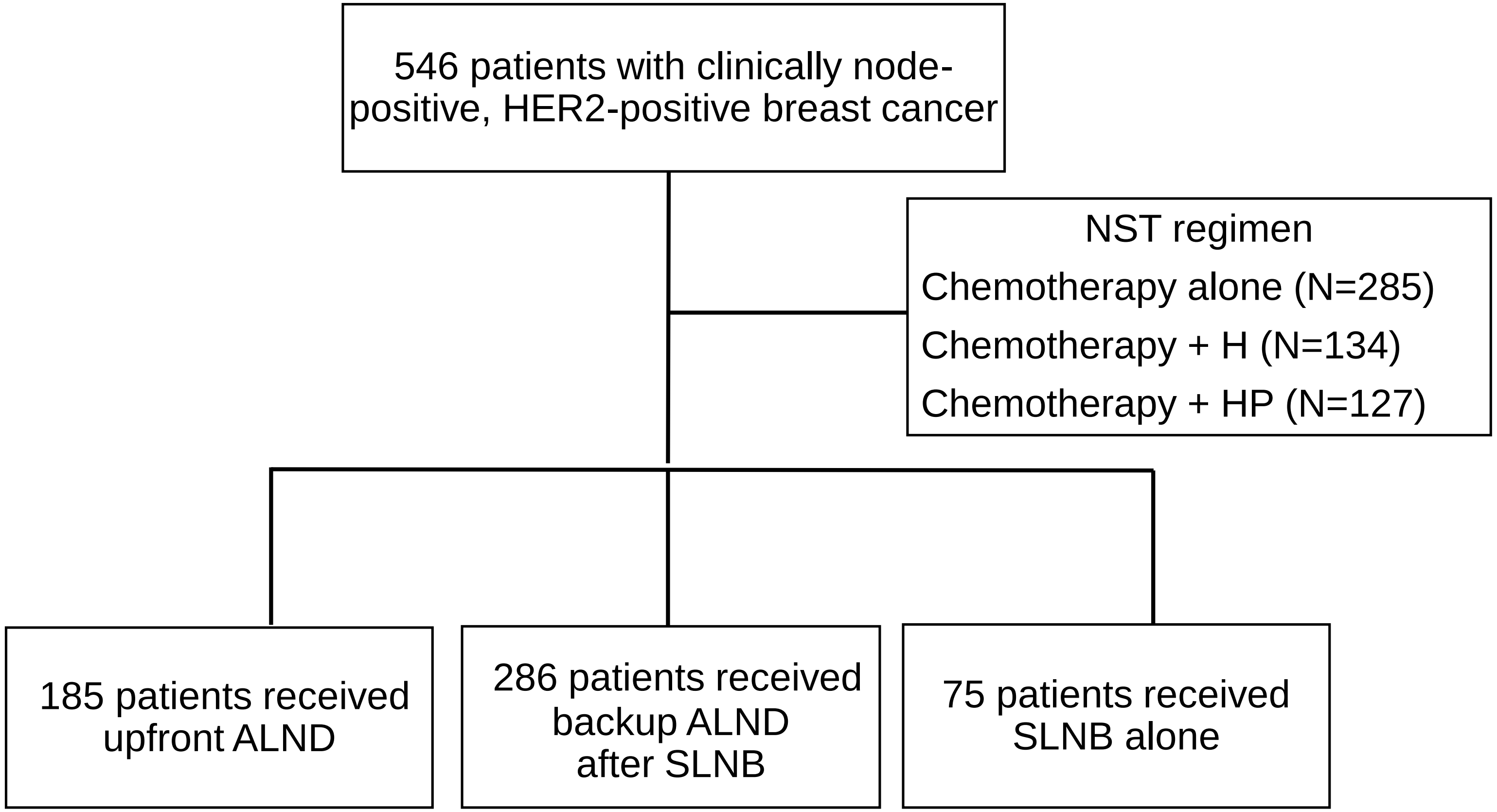

Abbreviations: NST, neoadjuvant systemic therapy; H, trastuzumab; HP, trastuzumab and pertuzumab

Figure S2. Axillary pathologic complete response rates in patients with biopsy-proven axillary metastasis

(A) All nodal stage (N = 376)

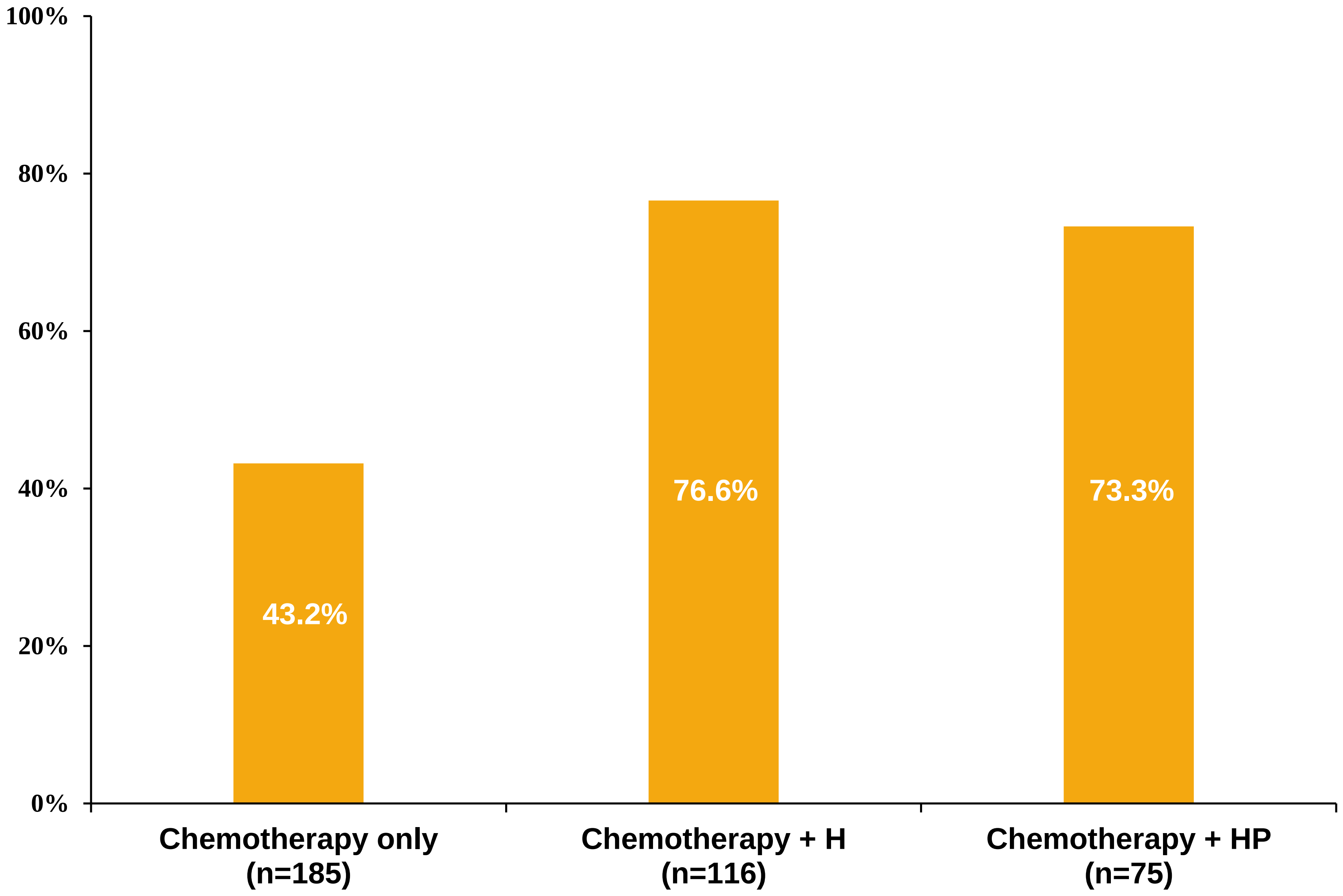

(B) Clinical N1 stage (N = 272)

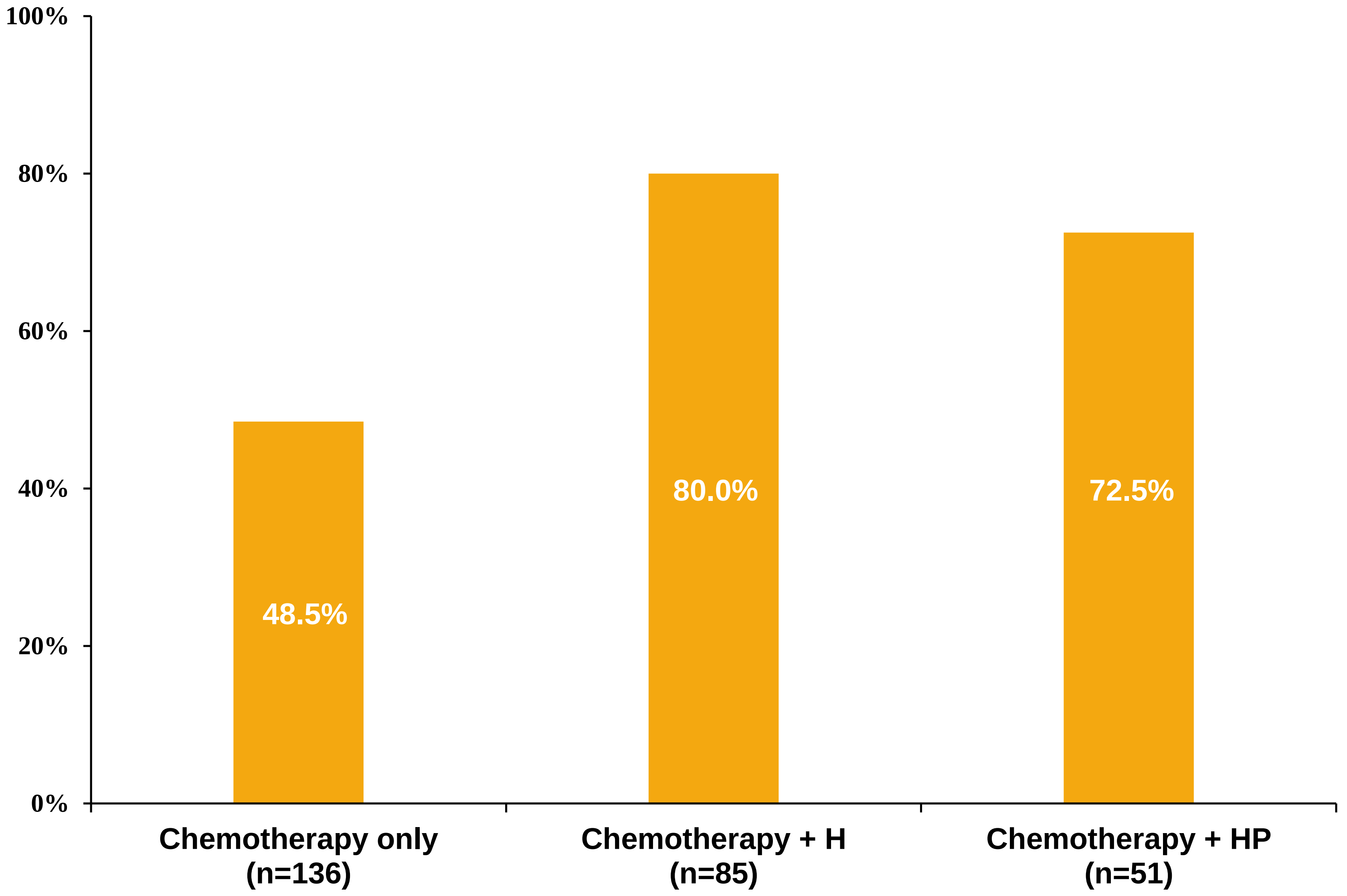

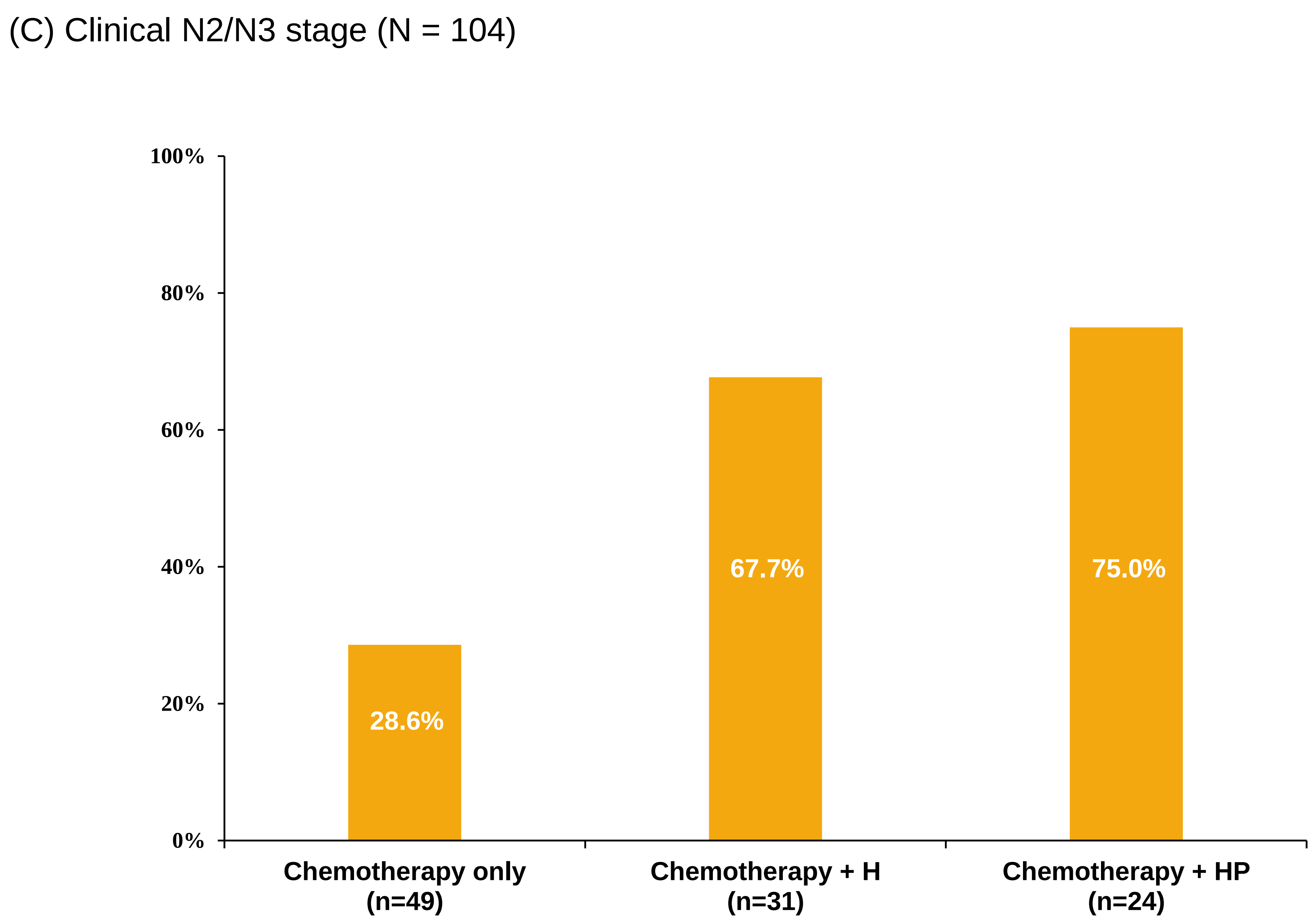
(C) Clinical N2/N3 stage (N = 104)
100%
80%
60%
40%
20%
0%
28.6%
67.7%
75.0%
Chemotherapy only
(n=49)
Chemotherapy + H
(n=31)
Chemotherapy + HP
(n=24)
